# Hospital and related resource costs associated with antimicrobial-resistant infections in Canada, 2019

**DOI:** 10.14745/ccdr.v48i1112a06

**Published:** 2022-11-03

**Authors:** Alan Diener, Hui Wang, Miriam Nkangu

**Affiliations:** 1 Policy Research, Economics, and Analytics Unit, Strategic Policy Branch, Health Canada, Ottawa, ON; 2 School of Epidemiology and Public Health, University of Ottawa, Ottawa, ON

**Keywords:** antimicrobial resistance, methicillin-resistant *Staphylococcus aureus*, *Clostridioides difficile*, hospital costs, health resources

## Abstract

**Background:**

Antimicrobial resistance (AMR) occurs when microorganisms become resistant to treatment by standard, or first-line, antibiotic drugs. These infections create an enormous burden on society due to longer hospital stays and increased morbidity and mortality, resulting in increased medical costs and foregone resources. The objective of this paper is to estimate the hospital costs associated with two of the most significant antibiotic-resistant organisms: methicillin-resistant *Staphylococcus aureus* (MRSA) and *Clostridioides difficile* (*C. difficile*), for Canada, for the year 2019, as well as the value of other resource use attributed to the lost production due to disability and premature mortality.

**Methods:**

The Discharge Abstract Database was employed for the analysis using a two-step process: first, the number of cases for each diagnosis was estimated; and then an average cost per case was derived, which was used to multiply the number of cases to obtain the total costs. Costs were derived using a regression model, accounting for demographic and other important confounding variables.

**Results:**

There were a total of 16,070 and 9,889 cases of *C. difficile* infections and MRSA infections, respectively, in Canada in 2019, resulting in an estimated 1,743 premature deaths. The majority of cases occurred in the older age groups. The hospital costs attributable to these infections were over $125 million, while the indirect resource costs were between $18.8 and $146.9 million.

**Conclusion:**

Quantifying the outcomes associated with antimicrobial-resistant infections provides valuable information for policymakers and is an essential first step in understanding the total economic impacts of AMR.

## Introduction

Antimicrobial resistance (AMR) is a serious and growing global public health threat in Canada and worldwide ((1–3)). Left unchecked, global economic costs could surpass $100 trillion by 2050, with Canada seeing a decrease in gross domestic product (GDP) upwards of $20 billion ((2–5)). Antimicrobial resistance occurs when microorganisms become resistant to treatment by standard, or first-line, antibiotic drugs. In recent years, more microbes have become resistant to current antibiotics, and few new antimicrobials have been brought to the market, resulting in increased illness due to previously treatable infections.

These infections create an enormous burden on society as patients face increased morbidity and mortality. In addition, AMR increases the burden on the healthcare system through increased lengths of stay in hospitals and the need for more expensive treatments and resources, which could be used to treat other conditions. With no effective treatment, antimicrobial-resistant infections persist, with a risk of spreading the infection to others.

Two of the most significant antibiotic-resistant organisms are methicillin-resistant *Staphylococcus aureus* (MRSA) and *Clostridioides difficile* (*C. difficile)*. Methicillin-resistant *Staphylococcus aureus* (*S. aureus*) can also be resistant to other first-line antibiotics such as oxacillin and cloxacillin. *Staphylococcus aureus* is present on the skin or mucosal surfaces of 20%–30% of the healthy population and is also known to cause systemic infection ((6)). Methicillin-resistant *S. aureus*, a specific type of staph bacteria, can be present on the skin or mucosal surfaces of both healthy populations and hospitalized patients, as well as on environmental surfaces, and can enter the body through broken areas in the skin, respiratory tract, surgical sites and/or open wounds and intravenous catheters, and can cause severe and sometimes fatal infections in the hospital setting. *Clostridioides difficile* is an important healthcare-associated infection that causes significant morbidity and mortality. It is the most common cause of infectious diarrhea in hospitals and can range from asymptomatic to life-threatening. Most cases occur in patients who are elderly and who have other underlying medical conditions. It spreads rapidly in healthcare settings by direct contact because it is naturally resistant to many antimicrobials used to treat other infections, and *C. difficile* spores in the environment tend to be resistant to commonly used disinfectants ((7)).

In addition to the direct medical costs, antimicrobial-resistant infections result in other foregone resources due to decreased production resulting from disability and premature mortality. If increases in AMR continue, the future burden associated with AMR may also increase significantly through its impact on the overall healthcare system. For example, as Smith and Coast (2012) noted, if antimicrobial resistance were to continue unchecked, we may face a world in which there is no longer any effective antibiotics available for situations in which they are currently routinely used ((8)).

Currently, there are few methodologically sound, comprehensive and comparable cost studies of AMR. Recent systematic reviews focusing on the costs of AMR found a wide variation in results due to the methodologies employed, type of resistance studied and the cost components included ((8–10)). For example, Naylor *et al.* found that excess healthcare system costs ranged from insignificance to $1 billion per year, while the economic burden ranged from $21,832 per case to $3 trillion in GDP loss ((9)). **Table 1** summarizes the results from these systematic reviews and recent Canadian studies that focused on the economic burden of AMR ((11–14)). Of note is the large variation in cost estimates due to the aforementioned reasons (all monetary costs were converted to 2019 Canadian dollars using Purchasing Power Parity values and inflated accordingly).

**Table 1:** Results of selected antimicrobial resistance economic burden studies

Reference (year of publication)	Region	Type of infection	Type of study	Estimated costs^a^
Smith and Coast (2013)	International	AMR in general	Systematic review	$5 to greater than $74,000 per patient episode
Levy *et al.* (2015)	Canada	*C. difficile*	Economic model using multiple data sources	$291 million in hospital costs $13 million in community medical costs $11 million in lost productivity
Thampi *et al.* (2015)	Ontario, Canada	MRSA	Multi-centre costing study	$14,100 direct costs per hospital patient
Zhang *et al.* (2016)	United States	*C. difficile*	Meta-analysis	$28,756 per patient
Naylor *et al.* (2018)	International	AMR in general	Systematic review	Healthcare system costs: up to $1 billion per year Economic burden: $29,595 per case to over $3 trillion in GDP losses
Canadian Council of Academies (2018)	Canada	AMR in general	Review of selected Canadian studies	$16,979 per MRSA patient $18,773 per AMR patient $1.5 billion in total AMR hospital costs
Zhen *et al.* (2019)	International	MRSA	Systematic review	$9,998 to $242,599 per patient

Abbreviations: AMR, antimicrobial resistance; *C. difficile*, *Clostridioides difficile*; GDP, gross domestic product; MRSA, methicillin-resistant *Staphylococcus aureus*

^a^ 2019 Canadian dollars

The Council of Canadian Academies (CCA) recently estimated the current and future health, social and economic impacts of AMR in Canada ((2)). Based on a review of several Canadian studies, the authors estimated an average cost of $16,280 per MRSA patient. Examining cost studies of other antimicrobial-resistant infections, the CCA estimated an average hospital case of AMR cost of $18,000. The studies included in the CCA analysis tended to be small-scale studies, many of which included data from only one or two hospital settings. Based on these cost estimates, total AMR hospital costs were estimated to be $1.4 billion in 2018. By 2050, AMR is projected to cost the Canadian healthcare system $6 billion at the current infection rate. Additionally, the report estimated that the cumulative loss in GDP due to AMR from 2018 to 2050 would be $268 billion if there were no changes to the current infection rate.

Notwithstanding the important concerns of researchers such as Smith and Coast, who warn that unless AMR is properly addressed, we are headed to a drastically different healthcare system than the one with which we are familiar, accurate estimates of the current overall burden of AMR are needed by policymakers. It is important to properly understand the current situation from which projections and modelling of future costs associated with AMR can be based. Valid data on the costs related to AMR in Canada would provide valuable information on the magnitude of its burden, address gaps in data, and provide evidence and inputs for policy analysis.

The objective of this paper was to estimate the hospital costs and vale of lost production associated with antimicrobial-resistant infections, specifically MRSA and *C. difficile* infections, in Canada for 2019. The incidence of antimicrobial-resistant infections was based on diagnosis only, using administrative data; no distinction was made between healthcare-acquired and community-acquired infections. Antimicrobial-resistant infections caused by other bacteria were excluded due to the lack of valid and reliable data.

## Methods

### Data sources

The main data source employed in the analysis was the Discharge Abstract Database (DAD) from the Canadian Institute for Health Information, from 2010 to 2019. The DAD contains administrative data on hospital discharges, diagnoses and patient characteristics, facilities in all provinces and territories except Québec are required to report to DAD. In addition to employing the standard DAD variables, data on the cost of a standard hospital stay and on the resource intensity weight associated with each hospital discharge were obtained. This allowed for the estimation of costs associated with each discharge. The cost of a standard hospital stay provides a cost for the standard hospital patient, while the resource intensity weight allows for the cost to be adjusted based on the patients’ characteristics and diagnoses. All analyses were run for data from 2010 to 2019. The cross-sectional results were from the most recent year, 2019, while the remaining data provided a look at AMR in Canada over time. Analysis was limited to those 18 years of age and over due to the low incidence in younger age groups.

While administrative rather than surveillance data were employed in the analysis, a recent study found that the DAD performed exceptionally well in identifying MRSA cases compared to surveillance data in Ontario and Alberta (r=0.79 for Ontario, r=0.92 for Alberta for overall, MRSA infections and r=0.95 for bloodstream MRSA infections in Ontario) ((15)). Thus, we are confident that the incidence rates produced using the DAD were valid estimates.

For each separation recorded, the DAD contains up to twenty-five possible diagnoses according to the tenth revision of the International Classification of Diseases (ICD-10) codes. Each record notes the most responsible diagnosis (MRDX), defined as “the diagnosis or condition that can be described as being most responsible for the patient’s stay in hospital. If there is more than one such condition, the one held most responsible for the greatest portion of the length of stay (LOS) or greatest use of resources is selected.” ((16)). All other diagnoses (up to twenty-four) were considered secondary diagnoses. For this analysis, all cases of MRSA and *C. difficile* infections (CDI) were identified (see **Table 2** for the specific ICD-10 codes employed in the analysis).

**Table 2:** ICD-10 codes employed to identify *Clostridioides difficile* infections and methicillin-resistant *Staphylococcus aureus* infections

Diagnosis	ICD-10 code(s)
CDI	A04.7
MRSA, non-BSI	B95.6 (*S. aureus*) and U82.1 (methicillin resistance) and in the same cluster^a^
MRSA, BSI	B95.6 (Staph Aureus) and U82.1 (methicillin resistance) and A49 (bloodstream infection), and in the same cluster or A41.0 (Sepsis due to Staphylococcus) and U82.1 (methicillin resistance) and in the same cluster^a^

Abbreviations: BSI, bloodstream infection; CDI, *Clostridioides difficile* infection; MRSA, methicillin-resistant *Staphylococcus aureus*

^a^ Starting in 2009, the Discharge Abstract Database included a variable noting which diagnoses are related by showing them in the same cluster. For an individual to have a methicillin-resistant infection, they must possess both the methicillin resistance and the infection diagnoses, and both diagnoses must be in the same cluster

As the DAD does not include data from the province of Québec, age-adjusted values for costs and mortality for Québec were estimated, based on the results obtained from the DAD, and included in the total values. Thus, all values of the total burden represent estimates for all of Canada. Results are presented in 2019 Canadian dollars.

### Incidence rates

Incidence and costs for MRSA infections were divided into bloodstream (BSI) and non-bloodstream (non-BSI) infections due to the differences in patients and treatment protocols. Prior to 2009, to be classified as a case of MRSA, the observation had to include both 1) a diagnosis of methicillin resistance and 2) a diagnosis of a *Staphylococcus* infection. In 2009, a cluster variable was introduced in the DAD to note whether the two diagnoses were related; thus, to be considered an MSRA case, the observation had to include both diagnoses and both diagnoses had to be identified as being within the same cluster. Incidence and costs for *C. difficile* diagnoses were estimated separately for those cases where *C. difficile* appeared as either a most responsible diagnosis or a secondary diagnosis (CDI MRDX and CDI non-MRDX, respectively.

### Hospital costs

Incremental costs—those costs associated with treating the conditions above and beyond the costs associated with the rest of that hospital stay—were estimated in two ways. Firstly, the average cost of patient stays with and without the diagnosis in question were estimated. The difference between the two estimates was then assumed to be the incremental costs attributable to the specific infection. The challenge with this approach is that the likelihood of an AMR infection increases with age, LOS, number of comorbidities and the reason for admission. Thus, unadjusted incremental costs derived in this manner are likely to overestimate the actual incremental costs that can be validly attributed to the presence of the infection only.

To account for the aforementioned confounding effects, the following regression model was employed to estimate the incremental costs associated with treating antimicrobial-resistant infections:

Cost = β_0_ + β_1_MRSA_non_BL + β_2_MRSA_BL + β_3_CDI_non_mrdx + β_4_Comorbidites

+ ∑γ_i_ISHMT_i_ + ∑λ_i_PROV_i_ + β_5_Sex + β_6_LOS + e

Where:

· Cost=the log of cost per discharge

· MRSA_non_BL=1 if non-bloodstream MRSA diagnosis present

· MRSA_BL=1 if bloodstream MRSA diagnosis present

· CDI_non_mrdx=1 if *C. difficile* diagnosis present as a comorbid condition

· Comorbidities=number of diagnosed comorbidities (excluding antimicrobial-resistant infections)

· ISHMT_i_=1 if the patient most responsible diagnosis is in International Short List of Hospital Morbidity Tabulation (ISHMT) code i (excluding the *C. difficile* code)

· PROV=a dummy for the province

· Sex=1, if female

· LOS=length of stay associated with the observation

The model employed included variables for most responsible diagnosis (to account for different reasons of admission), number of comorbidities (based on records in the DAD), sex, and province. The estimated beta coefficients were used to estimate the incremental costs associated with the infections. Specifically, the coefficients on the variables associated with infections (β_1_, β_2_, β_3_) were transformed to show the percentage increase in average costs that could be attributable to the infection (MRSA or *C. difficile*).

For those individuals with *C. difficile* as a secondary diagnosis or with a diagnosis of MRSA (which was always a secondary diagnosis), the incremental costs associated with that diagnosis were estimated.

Cost data are usually right-skewed, as costs cannot be negative and most of the observations are close to zero, with several observations having relatively high costs. Thus, a log-linear model was employed, allowing for a much better fit. The resulting beta coefficients, once transformed, can be interpreted as the incremental costs attributable to the presence of either MRSA or *C. difficile* infections, respectively, accounting for the age, diagnosis, sex, comorbidities and other relevant factors. Separate regressions were run by age group to account for differences by age. Once the incremental cost has been estimated, the cost is multiplied by the number of cases for that diagnosis. For patients classified as having *C. difficile* as a most responsible diagnosis, all costs associated with that hospital stay were employed.

The most responsible diagnosis, for each observation, was coded according to ISHMT. The ISHMT is a classification system based on ICD-10 Chapters, and it further breaks down the ICD Chapters into a total of 130 diagnostic categories. The ISHMT codes were employed to define the diagnoses as they represent a manageable number of well-defined diagnoses, while still being granular enough to be meaningful.

### Mortality estimates

To estimate the value of lost production due to premature mortality attributable to antimicrobial-resistant infections, it was necessary to estimate the increased mortality attributable to the infections employed in the analysis. While *C. difficile* is a possible listed cause of death, deaths attributable to MRSA infections are coded otherwise, making it difficult to obtain valid and reliable estimates on the number of deaths attributable to MRSA ((17)). Separate logistic regression with a binary variable of whether the patient died or was discharged from the hospital was used to estimate the mortality rate for each of the five age groups, namely 18–34, 35–54, 55–64, 65–74 and 75 years of age and older. The coefficients from such regression produce the log-odds, from which it was possible to estimate odd ratios for the mortality rates associated with each infection. Specifically, the following model was implemented:

Dead = β_0_ + β_1_MRSA_non_BL + β_2_MRSA_BL + β_3_CDI_non_mrdx + β_4_Comorbidites

+ ∑γ_i_ISHMT_i_ + ∑λ_i_PROV_i_ + β_5_Sex + β_6_LOS + e

Where:

· Dead=1 if patient died, 0 otherwise

· All other variables were previously defined

To estimate the total number of deaths attributable to each type of AMR infection, the age-specific death rate for all discharged patients and the number of AMR-specific infected patients were obtained from the data. Then, the infection-specific death rate can be calculated by multiplying the odds ratio of the specific infection and the overall death rate. Lastly, the estimated number of deaths for the infection can be estimated by multiplying the infection-specific death rate and the number of infections in the age group.

### Value of lost production

To obtain a more complete estimate of the economic burden, the value of the lost production, for both disability and premature mortality, attributable to antimicrobial-resistant infections was also estimated. Two approaches are generally employed to estimate production losses in cost of illness studies: the friction cost approach and the human capital approach ((18)). This friction cost approach assumes that a deceased worker will eventually be replaced by individuals currently in the pool of unemployed workers once those seeking employment are lined up with an employer currently offering employment (i.e. the friction period), with three months being a common time period employed ((19)). In contrast, the human capital approach measures the value of foregone gross lifetime earnings resulting in significantly larger estimates; that is, the human capital approach assumes that an individual’s production is lost for their entire working life. Given the ongoing debate on the appropriate method, and general higher rates of unemployment (the friction cost approach was originally proposed during periods of high unemployment), both methods were employed to increase the comparability of the results.

The length of time absent from work due to absenteeism or premature mortality was estimated using both approaches and was then multiplied by an average wage rate. The incremental LOS in hospitals attributable to these infections was estimated based on the previously estimated incremental costs to derive the amount of time missed due to absenteeism. Time missed from work was multiplied by age-specific average earnings (as a proxy for the marginal product). The average income and employment rate for persons aged 15 years of age and older were obtained from Statistics Canada ((20,21)).

## Results

### Incidence rates

**Figure 1:**
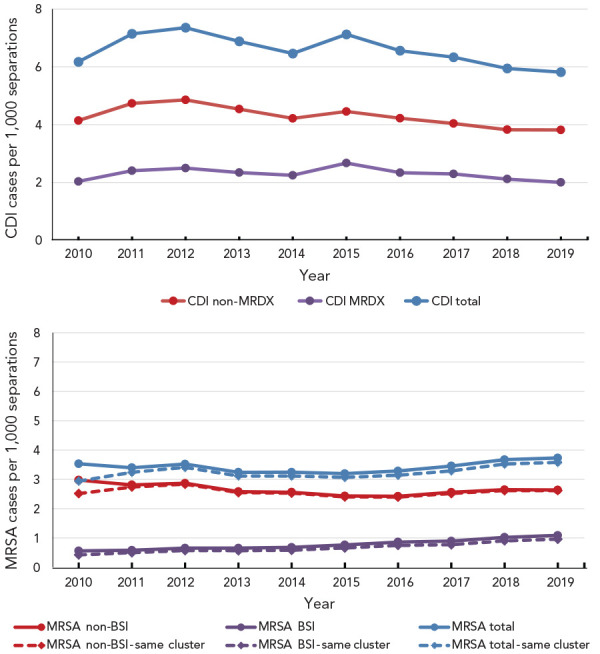
Incidence of *Clostridioides difficile* infections and methicillin-resistant *Staphylococcus aureus* infections, Canada^a^, 2010–2019 Abbreviations: BSI, bloodstream infection; CDI, *Clostridioides difficile* infections; MRDX, most responsible diagnosis; MRSA, methicillin-resistant *Staphylococcus aureus* ^a^ Excludes data from Québec

**Figure 1** shows the incidence of *C. difficile* and MRSA infections from 2010 to 2019. The incidence of *C. difficile* infections has fallen since 2015, from 7.1 cases per 1,000 hospital separations to 5.8 cases per 1,000 hospital separations in 2019 (2.0 as a most responsible diagnosis and 3.8 as a secondary diagnosis). When examining the methicillin-resistant and *Staphylococcus* infection diagnoses, it was observed that, in 2010 (the first year after the cluster variable was introduced), all cases had both a diagnosis of methicillin resistance and a *Staphylococcus* infection, and only 85% were in the same cluster. The majority (76%) of infections diagnosed as both methicillin-resistant and having a bloodstream *Staphylococcus* infection were also within the same cluster. In 2011, the percentage changed to 97% and 85%, and by 2019 the percentages stabilized at 99% and 88%, respectively. It likely took some time for the coding to be applied appropriately. In 2019, the overall rate for MRSA infections was 3.6 per 1,000 separations—2.6 for non-BSI and 1.0 for MRSA-BSIs. Note that bloodstream MRSA infections have increased steadily since 2010 and more than doubled between 2010 and 2019; from 0.4 to 1.0 cases per 1,000 hospital separations. These findings are consistent with the results of Canadian surveillance studies; however, due to differences in methodologies, results are not directly comparable ((1,7)).

In 2019 there were over 2.1 million hospital separations included in the DAD. **Table 3** presents summary statistics for the overall sample and individuals with either MRSA infections or CDI. Patients with any type of infection had a much longer average LOS; however, it should be noted that the difference between the average LOS for the entire sample and those with antimicrobial-resistant infections is attributable to many factors. Those with *C. difficile* infections tended to be older, and those with MRSA tended to be younger than the entire sample. While the average age for the entire sample increased over the study period, the average age of those with these infections decreased slightly. Males were more likely than females to have been diagnosed with an MRSA infection. **Table 4** shows the incidence rates for the antimicrobial-resistant infections by age group. Not surprisingly, those 75 years of age and over had the highest overall rates, except MRSA infections peaked for those 35–54 years of age.

**Table 3:** Summary statistics and incidence rates of antimicrobial-resistant infections, Canada^a^, 2019

Type of infection	Incidence (cases per 1,000 separations)	Percent female	Average LOS (days)	Average age (years)
Entire sample	N/A	57.0%	7.6	59.5
No infection	N/A	57.0%	7.5	59.5
CDI (MRDX) as most responsible diagnosis	2.00	58.9%	11.7	70.6
CDI, as non-MRDX	3.82	51.1%	31.7	68.8
CDI (total)	5.82	53.8%	24.8	69.4
MRSA non-BSI	2.62	40.5%	22.6	58.4
MRSA, BSI	0.96	38.6%	25.4	57.5
MRSA (total)	3.58	40.0%	23.3	58.2

Abbreviations: BSI, bloodstream infections; CDI, *Clostridioides difficile* infections; LOS, length of stay; MRDX, most responsible diagnosis; MRSA, methicillin-resistant *Staphylococcus aureus*, N/A, not applicable

^a^ Excludes data from Québec

**Table 4:** Incidence rates of antimicrobial-resistant infections, by age group, Canada^a^, 2019 (cases per 1,000 discharges)

Age group (years)	CDI, MRDX	CDI, non-MRDX	CDI, total	MRSA, non-BSI	MRSA, BSI	MRSA, total
18–34	0.537	0.994	1.531	1.970	0.758	2.728
35–54	1.119	2.492	3.611	3.805	1.455	5.261
55–64	1.929	4.315	6.244	3.055	1.141	4.195
65–74	2.415	5.028	7.443	2.509	0.909	3.418
75 and older	3.312	5.472	8.785	2.105	0.701	2.806
Total	2.002	3.816	5.818	2.621	0.960	3.580

Abbreviations: BSI, bloodstream infection; CDI, *Clostridioides difficile* infections; MRDX, most responsible diagnosis; MRSA, methicillin-resistant *Staphylococcus aureus*

^a^ Excludes data from Québec

### Hospital costs

The unadjusted costs were relatively high as expected and ranged from over $19,000 per patient (MRSA, non-BSI) to over $30,000 per patient (CDI). As previously noted, this is likely due to those with AMR having longer, more resource intensive, lengths of stay due to other characteristics. To derive the adjusted incremental costs, separate regressions were run for each age group (the main regression results are presented in **Appendix**, **Table A1** and **Table A2**). **Table 5** presents incremental cost estimates by age group). The average incremental costs across all age groups were $2,301 and $3,654 for non-BSI MRSA cases and BSI MRSA cases, respectively, resulting in a total hospital cost of MRSA estimated to be $24.4 million. For *C. difficile*, the average cost of patients having a most responsible diagnosis was $11,056 per patient and the incremental costs associated with a secondary *C. difficile* diagnosis was $3,749. Total hospital costs associated with *C. difficile* were estimated at $100.7 million.

**Table 5:** Hospital costs for antimicrobial-resistant infection per patient, by age group, Canada, 2019

Age group (years)	All diagnoses (average cost)	CDI, MRDX (incremental cost)	CDI, non-MRDX (incremental cost)	MRSA, non-BSI (incremental cost)	MRSA, BSI (incremental cost)
18–34	$5,251	$7,297	$2,806	$1,411	$1,828
35–54	$8,001	$7,866	$3,883	$1,694	$2,589
55–64	$10,785	$10,153	$3,731	$2,271	$3,022
65–74	$11,414	$12,389	$4,057	$2,309	$5,006
75 and older	$12,098	$11,806	$3,641	$2,408	$5,802
Average	$9,721	$11,056	$3,479	$2,031	$3,654

Abbreviations: BSI, bloodstream infection; CDI, *Clostridioides difficile* infections; MRDX, most responsible diagnosis; MRSA, methicillin-resistant *Staphylococcus aureus*

### Mortality estimates

The hospital separations provide the discharge disposition information; however, it was not specified whether a patient died in or outside the hospital. According to the DAD, the observed number of deaths with *C. difficile,* MRSA non-BSI, and MRSA BSI infection were 1,455, 353, and 351, respectively. As there is no cause of death for these patients noted in the data, the mortality might be due to other competing risks such as comorbidities or aging, instead of AMR infection alone.

To prevent the overestimation of AMR-related mortality, logistic regressions were conducted for the patients in each age group to estimate the death rates attributable to the infections, adjusted for sex, number of comorbidities and ISHMT diagnostic group. The results clearly showed a positive relationship between the number of deaths and the age of the patients. Table A2 presents the odds ratios obtained from the regression results, and **Table 6** shows the number of estimated deaths attributable to *C. difficile* and MRSA infections, for all of Canada. According to the estimates, the number of deaths attributable to *C. difficile,* MRSA non-BSI, and MRSA BSI was 1309, 257, and 177, respectively. The majority of the estimated deaths, near 70%, occurred among those aged 75 years and older.

**Table 6:** Estimated mortality by age group attributable to antimicrobial-resistant infections, Canada^a^, 2019

Type of infection	Age (years)					Total mortality
18–34	35–54	55–64	65–74	75 and older
CDI, any	2	28	98	206	975	1,309
MRSA, non-BSI	2	23	34	54	144	257
MRSA, BSI	2	17	25	49	84	177
Total	6	68	157	309	1,203	1,743

Abbreviations: BSI, bloodstream infection; CDI, *Clostridioides difficile* infections; MRDX, most responsible diagnosis; MRSA, methicillin-resistant *Staphylococcus aureus*

^a^ Excludes data from Québec

### Value of lost production

**Table 7** shows the incremental LOS associated with antimicrobial-resistant infections. The average LOS was multiplied by the number of cases, the average wage rate, and the employment rate to obtain the value of lost production due to morbidity, which totalled $5.6 million. The value of lost production due to premature mortality was estimated at $13.2 million using the friction cost approach, and $141.4 million using the human capital approach. This is consistent with other findings. The value of lost production is greatest for those aged 35 to 64 years old, resulting from higher earnings and employment in those age groups.

**Table 7:** Incremental length of stay associated with antimicrobial-resistant infections, Canada^a^, 2019

Type of infection	Age (years)				
	18–34	35–54	55–64	65–74	75 and older
CDI, MRDX^b^	6.9	7.5	9.3	12.6	13.5
CDI, non-MRDX	1.9	2.6	2.5	3.0	3.5
MRSA, non-BSI	1.0	1.1	1.5	1.7	2.3
MRSA, BSI	1.3	1.7	2.0	3.6	5.5

Abbreviations: BSI, bloodstream infection; CDI, *Clostridioides difficile* infections; MRDX, most responsible diagnosis; MRSA, methicillin-resistant *Staphylococcus aureus*

^a^ Excludes data from Québec

^b^ For CDI, MRDX the values refer to the average length of stay

### Total costs

**Table 8** summarizes the increased burden in terms of mortality and economic costs associated with antimicrobial-resistant infections in Canada in 2019. Antimicrobial-resistant infections resulted in 1,743 extra deaths and accounted for between $143.8 million and $272 million in total economic costs.

**Table 8:** Burden associated with antimicrobial-resistant infections, Canada^a^, 2019

Type of infection	Number of cases	Increased mortality	Hospital costs^b^	Lost production^b^ (disability)	Lost production^b^ (premature mortality)		Total costs^b^
					FCM	HCM	
CDI, any	16,070	1,309	$100.65	$3.99	$9.92	$66.90	$114.56–$171.54
MRSA, non-BSI	7,238	257	$14.70	$0.93	$1.95	$42.62	$17.5–$58.26
MRSA, BSI	2,651	177	$9.69	$0.64	$1.34	$31.82	$11.6–$42.15
Total	25,959	1,743	$125.04	$5.56	$13.22	$141.35	$143.8–$271.95

Abbreviations: BSI, bloodstream infection; CDI, *Clostridioides difficile* infections; FCM, friction cost method; HCM, human capital method; MRSA, methicillin-resistant *Staphylococcus aureus*

^a^ Estimates include Québec

^b^ 2019 Canadian dollars, in millions

## Discussion

There were an estimated 16,070 and 9,989 cases of *C. difficile* and MRSA infections, respectively, in Canada in 2019, resulting in an estimated 1,743 premature deaths. The majority of cases occurred in the older age groups, and nearly 70% of the premature deaths occurred among those aged 75 years and older. The annual hospital-related costs were over $125 million, while the value of lost production was estimated to be between $18.8 million and $146.9 million; total economic costs were between $143.8 million $272 million. Given the assumptions employed and noting that only two types of antimicrobial-resistant infections were incorporated in the analysis, these results can be considered lower values of the economic burden of antimicrobial-resistant infections in Canada.

The estimates for LOS, attributable mortality and incremental costs were consistent with those found in the literature, although at the low end. This finding is not unexpected, given that the methodology employed in the estimation of hospital costs was likely to produce conservative estimates. In addition, the analysis attempted to account for factors that may influence the risk of antimicrobial-resistant infections and would affect total costs, including age, LOS, number of comorbidities and the most responsible diagnosis. Differences in per patient hospital costs were likely due to estimating incremental, rather than average costs.

Direct comparisons with the previous literature are challenging due to the wide range of outcomes included, perspective, and methodologies employed. Naylor *et al.* ((9)) noted that much of the previous evidence on the economic burden of AMR did not employ established health economic modelling techniques; they produced recommendations for AMR economic burden research, which we attempted to follow. This included using a representative population sample, taking into account confounding variables (including comorbidities and age), describing the data employed and how rates were derived, and clearly describing the model employed.

### Limitations

While attempting to consider many of the covariates related to antimicrobial-resistant infections, the analysis had several limitations. As previously noted, the analysis did not distinguish between health care-acquired and community-acquired infections. The differences between these two patient groups may affect overall outcomes and ideally should be accounted for. In addition, the data employed focussed on hospital separations instead of actual individuals. Thus, it was not possible to account for possible readmissions. Having such data would allow a better estimate of overall AMR cases rather than episodes. Related to the latter point, antimicrobial-resistant infections may result in long-term health impacts and thus costs. For example, Nanwa *et al.* conducted a longitudinal, matched cohort, study in Ontario, Canada, that estimated the three-year costs associated with CDI finding that the costs were greater than $31,000 and $37,000 (2014 CDN$) for non-elective and elective admission patients ((22)).

## Conclusion

Quantifying the outcomes associated with antimicrobial-resistant infections provides valuable information for policymakers and is an essential first step in understanding the total economic impacts of AMR. Quantifying these outcomes is also an important input that can be used in economic evaluations of policies to reduce the future impacts of AMR.

## Appendix List of tables

Table A1: Incremental cost regression results

Table A2: Mortality regression results

**Table A1:** Incremental cost regression results

Age group	Independent variable	Coefficient^a^	Standard error	T-statistic	Regression statistics
18–34	*C. difficile*, non-MRDX	0.4282	0.0221	19.37	Number of observations: 398,445 F–statistic: 3,964.2 Probability >F: <.0001 R-squared: 0.573 Adj. R-squared: 0.573
	MRSA, non-BSI	0.2380	0.0159	14.93	
	MRSA, BSI	0.2987	0.0256	11.68	
	Female	-0.0363	0.0023	-15.93	
	Length of stay	0.0301	0.0001	323.55	
	Comorbidities	0.0777	0.0003	223.69	
	Constant	7.8083	0.0162	482.12	
35–54	*C. difficile*, non-MRDX	0.3956	0.0167	23.70	Number of observations: 401,292 F–statistic: 3,965.0 Probability >F: <.0001 R-squared: 0.575 Adj. R-squared: 0.575
	MRSA, non-BSI	0.1921	0.0137	14.04	
	MRSA, BSI	0.2803	0.0220	12.75	
	Female	-0.0213	0.0020	-10.74	
	Length of stay	0.0274	0.0001	340.48	
	Comorbidities	0.0807	0.0004	230.28	
	Constant	7.7870	0.0186	417.89	
55–64	*C. difficile*, non-MRDX	0.2971	0.0153	19.47	Number of observations: 326,065 F–statistic: 3,192.3 Probability >F: <.0001 R-squared: 0.566 Adj. R-squared: 0.566
	MRSA, non-BSI	0.1911	0.0181	10.53	
	MRSA, BSI	0.2470	0.0296	8.35	
	Female	-0.0100	0.0021	-4.80	
	Length of stay	0.0248	0.0001	326.28	
	Comorbidities	0.0815	0.0004	223.42	
	Constant	7.8015	0.0248	315.15	
65–74	*C. difficile*, non-MRDX	0.3041	0.0126	24.08	Number of observations: 403,732 F–statistic: 4,221.8 Probability >F: <.0001 R-squared: 0.578 Adj. R-squared: 0.578
	MRSA, non-BSI	0.1842	0.0178	10.33	
	MRSA, BSI	0.3637	0.0296	12.30	
	Female	-0.0012	0.0018	-0.65	
	Length of stay	0.0238	0.0001	388.35	
	Comorbidities	0.0796	0.0003	262.67	
	Constant	7.9524	0.0240	331.05	
75 and older	*C. difficile*, non-MRDX	0.2631	0.0099	26.63	Number of observations: 606,518 F–statistic: 6,794.8 Probability >F: <.0001 R-squared: 0.595 Adj. R-squared: 0.595
	MRSA, non-BSI	0.1815	0.0159	11.44	
	MRSA, BSI	0.3917	0.0275	14.25	
	Female	0.0065	0.0015	4.34	
	Length of stay	0.0215	0.0000	559.10	
	Comorbidities	0.0760	0.0002	328.88	
	Constant	8.3914	0.0243	345.42	

Abbreviations: Adj., adjusted; BSI, bloodstream infection; CDI, *Clostridioides difficile*; MRDX, most responsible diagnosis; MRSA, methicillin-resistant *Staphylococcus aureus*

^a^ All coefficients were statistically significant at the 1% confidence level except for sex in the 65–74 age group. Coefficients for the provincial/territorial and International Short List of Hospital Morbidity Tabulation dummies are not shown. Source: Health Canada, Policy Research, Economics and Analytics

**Table A2:** Mortality regression results

Age group	Independent variable	Odds ratio	Standard error	Wald Chi-square	Regression statistics
18–34	*C. difficile*, any	1.095	0.303	0.090	Number of observations: 398,445 Likelihood ratio: 6,205.05 Probability >F: <0.0001 R-squared: 0.016 Max-rescaled R-square: 0.4071
	MRSA, non-BSI	0.684	0.371	1.049	
	MRSA, BSI	2.123	0.285	6.956	
35–54	*C. difficile*, any	1.178	0.137	1.430	Number of observations: 401,292 Likelihood ratio: 19,382.09 Model significance: <0.0001 R-squared: 0.0472 Max-rescaled R-square: 0.3699
	MRSA, non-BSI	0.901	0.181	0.330	
	MRSA, BSI	1.815	0.157	14.367	
55–64	*C. difficile*, any	1.179	0.098	2.864	Number of observations: 326,065 Likelihood ratio: 27,465.87 Model significance: <0.0001 R-squared: 0.0808 Max-rescaled R-square: 0.3308
	MRSA, non-BSI	0.838	0.172	1.059	
	MRSA, BSI	1.595	0.167	7.805	
65–74	*C. difficile*, any	1.113	0.074	2.068	Number of observations: 403,732 Likelihood ratio: 44,814.01 Model significance: <0.0001 R-squared: 0.1051 Max-rescaled R-square: 0.3312
	MRSA, non-BSI	0.884	0.129	0.904	
	MRSA, BSI	2.172	0.143	29.419	
75 and older	*C. difficile*, any	1.584	0.047	95.419	Number of observations: 606,518 Likelihood ratio: 84,928.33 Model significance: <0.0001 R-squared: 0.1307 Max-rescaled R-square: 0.2888
	MRSA, non-BSI	0.973	0.095	0.082	
	MRSA, BSI	1.715	0.126	18.212	

Abbreviations: BSI, bloodstream infection; CDI, *Clostridioides difficile*; MRSA, methicillin-resistant *Staphylococcus aureus*

## References

[1] Public Health Agency of Canada. Canadian Antimicrobial Resistance Surveillance System - 2021Ottawa, ON: PHAC; 2022. https://www.canada.ca/en/public-health/services/publications/drugs-health-products/canadian-antimicrobial-resistance-surveillance-system-report-2021.html

[2] Council of Canadian Academies. When Antibiotics Fail. The Expert Panel on the Potential Socio-Economic Impacts of Antimicrobial Resistance in Canada. Ottawa, ON: CCA; 2019. https://cca-reports.ca/reports/the-potential-socio-economic-impacts-of-antimicrobial-resistance-in-canada/

[3] Antimicrobial Resistance Collaborators. Global burden of bacterial antimicrobial resistance in 2019: a systematic analysis. Lancet 2022;399(10325):629–55. 10.1016/S0140-6736(21)02724-0 35065702 PMC8841637

[4] UK Government. Review on Antimicrobial Resistance. Antimicrobial resistance: tackling a crisis for the health and wealth of nations. London, UK: UK Government; 2014. https://amr-review.org/sites/default/files/AMR%20Review%20Paper%20-%20Tackling%20a%20crisis%20for%20the%20health%20and%20wealth%20of%20nations_1.pdf

[5] UK Government. Review on Antimicrobial Resistance. Tackling Drug Resistant Infections Globally: Final Report and Recommendations. London, UK: UK Government; 2014. https://amr-review.org/sites/default/files/160518_Final%20paper_with%20cover.pdf

[6] Lucet JC, Regnier B. Screening and decolonization: does methicillin-susceptible Staphylococcus aureus hold lessons for methicillin-resistant S. aureus? Clin Infect Dis 2010;51(5):585–90. 10.1086/655695 20662715

[7] Public Health Agency of Canada. Canadian Antimicrobial Resistance Surveillance System - 2017 Report. Ottawa, ON: PHAC; 2017. https://www.canada.ca/en/public-health/services/publications/drugs-health-products/canadian-antimicrobial-resistance-surveillance-system-2017-report-executive-summary.html

[8] Smith R, Coast J. The economic burden of antimicrobial resistance: Why it is more serious than current studies suggest. London School of Hygiene and Tropical Medicine: 2012. https://researchonline.lshtm.ac.uk/id/eprint/639028/1/DH_AMR_final_report_30-10-12_with_appendix.pdf

[9] Naylor NR, Atun R, Zhu N, Kulasabanathan K, Silva S, Chatterjee A, Knight GM, Robotham JV. Estimating the burden of antimicrobial resistance: a systematic literature review. Antimicrob Resist Infect Control 2018;7:58. 10.1186/s13756-018-0336-y 29713465 PMC5918775

[10] Smith R, Coast J. The true cost of antimicrobial resistance. BMJ 2013;346:f1493. https://www.bmj.com/content/346/bmj.f1493 10.1136/bmj.f1493 23479660

[11] Levy AR, Szabo SM, Lozano-Ortega G, Lloyd-Smith E, Leung V, Lawrence R, Romney MG. Incidence and Costs of Clostridium difficile Infections in Canada. Open Forum Infect Dis 2015;2(3):ofv076. 10.1093/ofid/ofv076 26191534 PMC4503917

[12] Thampi N, Showler A, Burry L, Bai AD, Steinberg M, Ricciuto DR, Bell CM, Morris AM. Multicenter study of health care cost of patients admitted to hospital with Staphylococcus aureus bacteremia: impact of length of stay and intensity of care. Am J Infect Control 2015;43(7):739–44. 10.1016/j.ajic.2015.01.031 25769617

[13] Zhang S, Palazuelos-Munoz S, Balsells EM, Nair H, Chit A, Kyaw MH. Cost of hospital management of Clostridium difficile infection in United States-a meta-analysis and modelling study. BMC Infect Dis 2016;16(1):447. 10.1186/s12879-016-1786-6 27562241 PMC5000548

[14] Zhen X, Lundborg CS, Sun X, Hu X, Dong H. Economic burden of antibiotic resistance in ESKAPE organisms: a systematic review. Antimicrob Resist Infect Control 2019;8:137. 10.1186/s13756-019-0590-7 31417673 PMC6692939

[15] Ramirez Mendoza JY, Daneman N, Elias MN, Amuah JE, Bush K, Couris CM, Leeb K. A Comparison of Administrative Data Versus Surveillance Data for Hospital-Associated Methicillin-Resistant Staphylococcus aureus Infections in Canadian Hospitals. Infect Control Hosp Epidemiol 2017;38(4):436–43. 10.1017/ice.2016.302 27995814

[16] Canadian Institute for Health Information. Canadian Coding Standards for Version 2012 ICD-10-CA and CCI. Ottawa, ON: CIHI; 2012. https://secure.cihi.ca/estore/productSeries.htm?pc=PCC189

[17] Dubberke ER, Gerding DN, Classen D, Arias KM, Podgorny K, Anderson DJ, Burstin H, Calfee DP, Coffin SE, Fraser V, Griffin FA, Gross P, Kaye KS, Klompas M, Lo E, Marschall J, Mermel LA, Nicolle L, Pegues DA, Perl TM, Saint S, Salgado CD, Weinstein RA, Wise R, Yokoe DS. Strategies to prevent clostridium difficile infections in acute care hospitals. Infect Control Hosp Epidemiol 2008;29 Suppl 1:S81–92. 10.1086/591065 18840091

[18] Pike J, Grosse SD. Friction cost estimates of productivity costs in cost-of-illness studies in comparison with human capital estimates: a review. Appl Health Econ Health Policy 2018;16(6):765–78. 10.1007/s40258-018-0416-4 30094591 PMC6467569

[19] Koopmanschap MA, Rutten FF, van Ineveld BM, van Roijen L. The friction cost method for measuring indirect costs of disease. J Health Econ 1995;14(2):171–89. 10.1016/0167-6296(94)00044-5 10154656

[20] Statistics Canada. Table 11-10-0239-01. Income of individuals by age group, sex and income source, Canada, provinces and selected census metropolitan areas. Ottawa, ON: StatCan; 2021. https://www150.statcan.gc.ca/t1/tbl1/en/tv.action?pid=1110023901

[21] Statistics Canada. Table 14-10-0020-01. Unemployment rate, participation rate and employment rate by educational attainment, annual. Ottawa, ON: StatCan; 2021. https://www150.statcan.gc.ca/t1/tbl1/en/tv.action?pid=1410002001

[22] Nanwa N, Kwong JC, Krahn M, Daneman N, Lu H, Austin PC, Govindarajan A, Rosella LC, Cadarette SM, Sander B. The Economic Burden of Hospital-Acquired Clostridium difficile Infection: A Population-Based Matched Cohort Study. Infect Control Hosp Epidemiol 2016;37(9):1068–78. 10.1017/ice.2016.122 27322606

